# Integration of Additive Manufacturing, Parametric Design, and Optimization of Parts Obtained by Fused Deposition Modeling (FDM). A Methodological Approach

**DOI:** 10.3390/polym12091993

**Published:** 2020-09-02

**Authors:** Amabel García-Dominguez, Juan Claver, Miguel A. Sebastián

**Affiliations:** Department of Manufacturing Engineering, Universidad Nacional de Educación a Distancia (UNED), 28040 Madrid, Spain; amabel.garcia@invi.uned.es (A.G.-D.); msebastian@ind.uned.es (M.A.S.)

**Keywords:** additive manufacturing, FDM, optimization, parametric design, infill optimization, mass customizing

## Abstract

The use of current computer tools in both manufacturing and design stages breaks with the traditional conception of productive process, including successive stages of projection, representation, and manufacturing. Designs can be programmed as problems to be solved by using computational tools based on complex algorithms to optimize and produce more effective solutions. Additive manufacturing technologies enhance these possibilities by providing great geometric freedom to the materialization phase. This work presents a design methodology for the optimization of parts produced by additive manufacturing and explores the synergies between additive manufacturing, parametric design, and optimization processes to guide their integration into the proposed methodology. By using Grasshopper, a visual programming application, a continuous data flow for parts optimization is defined. Parametric design tools support the structural optimization of the general geometry, the infill, and the shell structure to obtain lightweight designs. Thus, the final shapes are obtained as a result of the optimization process which starts from basic geometries, not from an initial design. The infill does not correspond to pre-established patterns, and its elements are sized in a non-uniform manner throughout the piece to respond to different local loads. Mass customization and Fused Deposition Modeling (FDM) systems represent contexts of special potential for this methodology.

## 1. Introduction

Additive manufacturing technologies [1,2] represent a paradigm shift from traditional manufacturing technologies [3,4,5,6,7]. Parts are obtained adding material layer upon layer until the final geometry is completed, what offers various advantages over traditional processes, such as obtaining the final geometry in a single process and the great geometric freedom that these technologies allow [1,8].

Restrictions imposed by productive processes must be taken into account when designing a product, since ignoring these limitations would lead to failure in the materialization phase. In this sense, additive manufacturing eliminates some restrictions in comparison to traditional manufacturing technologies, but they also have limitations regarding to technologies themselves and the influence of manufacturing parameters [9,10,11,12,13,14,15,16,17,18,19,20,21], materials behavior [21,22,23,24,25,26,27] or context aspects, such as the standardization of these kind of processes and products [9,28,29]. Additive manufacturing gives great freedom to designs, and it allows the obtaining of very complex geometries that were previously impossible, especially with regard to interior cavities. In addition, additive manufacturing also introduces the idea of infill, which offers new opportunities for those parts of the pieces which were understood as solid within traditional manufacturing scenarios and that currently represents a significant research field [30,31,32,33]. This possibility means using a smaller amount of material, what from a general point of view represents interesting opportunities such as cost savings in terms of material and time resources as well as the possibility to produce lightweight parts. However, approaches based on lightened structures also offer specific opportunities in different fields, such as load-bearing structures used in coated implants [34] or the manufacturing of personalized orthosis adapted to the anatomy of the patient and with advantages in terms of weight, ventilation and hygiene advantages [35]. Therefore, this context offers a wide range of design possibilities and even allows the definition of specific criteria or design strategies to additive manufacturing contexts [36,37,38,39].

Due to this higher level of geometric freedom, design optimization becomes more important [8], since the complexity of the geometry resulting from the optimization process is no longer a problem. In addition, on the other hand, the number of feasible solutions increases significantly, so selecting the most suitable alternatives becomes more complicated. Currently, the existing additive manufacturing technologies have achieved greater presence and recognition in the productive field, and at the same time design and optimization tools have achieved the capacity to respond to the new challenges that the new capacities of these productive technologies offer to designers.

Regarding the approach of the research carried out, the methodology presented in this work is based on three technologies and the synergies between them. Thus, it represents the simultaneous implementation of additive manufacturing technologies, parametric design tools, and optimization techniques. None of them alone could support this methodological proposal, which is the result of exploring the possibilities offered by their combined use. From this point of view, the authors consider that these types of approaches are also of interest in the field of teaching, as resources that can help students better understand some key aspects of each of the technologies considered, as well as helping them to explore the new application possibilities these technologies have in productive scenarios and the opportunities that each one offers professionals in their activity.

Thanks to the parametric design and the programming developed with Grasshoppers [40], parameters that represent key aspects and that help to achieve certain objectives are taken into consideration simultaneously. Thus, the mechanical resistance of the structures obtained from topology optimization both of geometry and infill structure, which represents the main mechanical behavior of the piece, are verified by applying Finite Element Analysis. However, the proposed methodology is not conceived as an absolute solution for all possible scenarios nor does it consider all the parameters and aspects that may have influence. The main contribution of the work carried out is to show an initial strategy for the integration of the three technologies, and based on the obtained results the authors consider this study functional and useful. However, the methodology can be implemented incorporating more specific aspects when applicable by adding new parameters to the programming, as it could be the case of parts that must withstand impacts.

The objective of this work is to define a continuous workflow by using parametric design able to obtain optimized designs for their production by additive manufacturing technologies. The aim of the methodology is to design lightweight pieces from its geometry, infill, and shell. In that sense, a relevant achievement of the present work is that the optimization problem is defined from the initial volume in a continuous data flow with no interruption after the topology optimization, therefore no redefining of the model is needed. On the other hand, this work represents one of the main results of the research carried out within the framework of the first author’s doctoral thesis [41], and it represents an important step in the fulfilment of one of the main objectives set in previous works [8] in relation to the optimization of the infill in a non-uniform manner throughout the piece. Thus, the infill structures are adapted to local loads, and this process is incorporated within the global methodology for the design of optimized parts to be obtained by additive manufacturing.

Although the designed methodology is applicable to any additive manufacturing technology, the case studies developed so far by the authors focus on small prototypes obtained using FDM (Fused Deposition Modeling) 3D printers. FDM technologies are of special interest for this work from different points of view. First, opened shell designs as the wireframed shell applied in the proposed methodology allow the removal of any required support material. Furthermore, the increasing access of FDM technology equipment encourage its massive interest and use providing technological tools for mass customization approaches. The influence of the materials and technologies used required some previous studies focused on these aspects [9,28].

## 2. Initial Considerations and Main Synergies of the Technologies Considered

For each technology, the basic concepts and the characteristics of greatest interest for this work are briefly introduced below, and their main synergies and opportunities for combined use are presented, composing a brief state of the art prior to the exposition of the proposed methodology.

### 2.1. Additive Manufacturing

**Figure 1:**
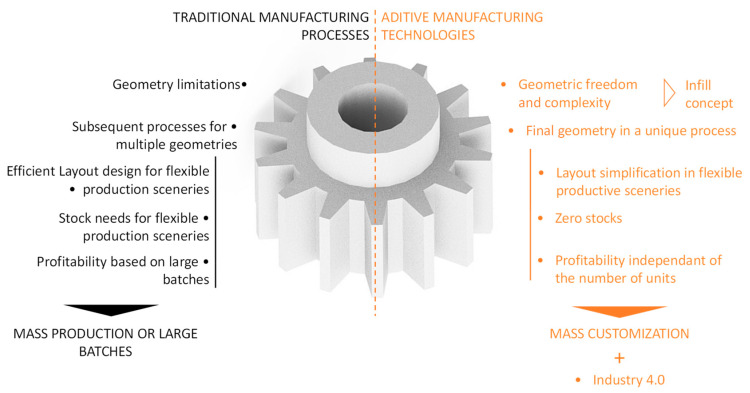
Main differences of additive manufacturing technologies compared to traditional manufacturing technologies.

As illustrated in Figure 1, additive manufacturing technologies offer, compared to traditional manufacturing technologies, some relevant advantages and opportunities. Additive manufacturing processes introduce enormous geometric freedom thanks to layer upon layer manufacturing, especially regarding interior cavities [3,36]. In fact, as previously stated, additive manufacturing processes involve the appearance of the concepts of shell and infill.

The possibility of designing infill patterns opens up new scenarios that where inconceivable before the irruption of additive manufacturing. By acting on the infill pattern, both on its geometry and on its density, the amount of material in the part is modified, which affects parameters such as the manufacturing time, the amount of material used, the cost and, of course, the lightness of the piece and its mechanical capacity. In this sense, this work goes further and instead of pre-established and continuous infill patterns, it proposes its optimization with a variable density throughout the piece. This aspect is considered one of the main contributions of the proposed methodology.

Moreover, the capability of obtaining a complex geometry in a single equipment and in a single process represents another advantage of these technologies. Product customization finds in these technologies a totally new scenario in which customization is not synonymous with high cost [42]. The concept of mass customization is not new, and it is a challenge that has already been attempted to be faced without the support of additive manufacturing [43,44,45]. However, when additive technologies are consolidated as manufacturing technologies, they will probably be the best way to achieve mass customization objectives [42,45,46]. In fact, additive manufacturing allows so many new possibilities and with so much impact that they make it possible to speak of a new paradigm in terms of mass customization [47]. A reality that opens many lines of application and business, and that in fields such as medicine and pharmaceutics, where additive manufacturing is already being widely applied [48,49,50,51,52,53,54,55,56], can be especially beneficial by offering personalized solutions tailored to patients.

Thus, today additive manufacturing represents a real alternative that is applied in very different productive contexts [57,58], what has been driving a great development in terms of increasingly efficient equipment and processes, as well as new materials [59,60,61]. Thus, additive manufacturing radically changes the productive paradigm, offering new scenarios and possibilities, both in relation to the agents involved and their roles and in the way in which products are thought and conceived [62,63,64,65,66]. In this sense, it highlights the impact of the low-cost alternatives for additive manufacturing systems, already regularly present in schools, small businesses, and even homes. Something that is already modifying the traditional relationship between designer, manufacturer, and consumer, where all these roles may even fall on the same person. A trend that could lead to converting these 3D printers into everyday objects in our homes, proposing new consumption structures [8].

### 2.2. Parametric Design

Unlike traditional CAD (Computer Aided Design) tools, which use closed scripts provided by the software developer for the formal representation of a solution previously conceived, parametric design approaches are based on a network of relations between variables where different values can be applied, producing different solutions to achieve the objectives. The work developed by Caetano et al. [67] carries out a significant review of recent advances as well as the terminology and definitions in regard to these kind of design strategies, and proposes his own definition of parametric design, which is understood as “an approach that describes a design symbolically based on the use of parameters”. In the context of the present work, it results of key importance the update of the solution generated when any parameter is modified, an aspect that parametric approaches allow and which in 1989 was included by Kalay [68] into the usual definition of parametric design.

The parametric design tools entail a new way of understanding design where it is necessary to decompose the problem into essential parameters of the project, as well as coding rules and protocols where any alteration of the parameters leads to a different model. At the same time, the development of new software offers the designer more and more flexibility and allows him to create his own action sequences through different programming languages, classifiable into two groups; those that describe routines in writing and those that do it visually. Parametric models based on visual programming can be represented in a general graph with a set of nodes, both operation (geometric or otherwise) and information, connected to each other by arrows that establish a workflow [69].

Presently, research about parametric design opportunities is really significant, as well as the application of these techniques into a wide range of industrial sectors. In that context it is possible to identify a great amount of studies based on this kind of design strategies and from very different contexts and approaches [70,71].

### 2.3. Design Optimization

Design optimization can be carried out through multicriteria analysis to aid decision-making or through multi-objective optimization. Multicriteria decision-making considers several possible solutions or alternatives and selects the most appropriate applying its criteria structure. Multi-objective optimization does not consider a finite number of alternatives, there are infinite solutions and they are represented by the variables of the problem delimited by the restrictions.

The calculation methods of the optimization problems look for the value of the variable or variables that maximize or minimize the objective function, which are the ones that obtain the highest or lowest value of the objective to be achieved and within the ranges determined by the constraints of the problem. In the case of multi-objective optimization problems, the objective functions are in conflict with each other [72]. If they were not, the optimization problem could be divided into single-optimization problems taking each of the values separately. This work focuses on the study of multi-objective optimization problems with the aim of avoiding predetermined formal solutions as far as possible. Various calculation methods are available in the proposed methodology and it will be convenient to apply one or the other depending on the design problem [41].

### 2.4. Opportunities and Synergies

#### 2.4.1. Mass Customization

Compared to serial and large batch manufacturing, the traditional concept of customization is associated with higher lead times and costs per unit [73]. Reducing these values represents the biggest challenge of mass customization [52], and the technologies integrated within the proposed methodology significantly contribute to these objectives. Additive manufacturing allow the rapid manufacturing of unique models at low cost [74] and, in that sense, FDM technologies and multi-material possibilities are productive scenarios of great potential [75], although still with limitations derived from the process and the materials [76]. Probably, the applications in the field of medicine represent the clearest example to understand the importance and potential of customization [56,77], but it is not the only one. Any ergonomic product is interesting from the point of view of mass customization [41,47], but really in all fields there are examples of applications that require unique and optimal solutions, construction industry for example [78]. Thus, the demand for personalized features without extra costs is an upward trend [79] in all types of products, even incorporating the customer in the design process [80,81,82].

Mass customization, as a concept, is in a process of continuous revision and evolution, from a system based on modular elements to a system based on the co-design of products by consumers [73,79,83,84,85], so companies need new studies, methods, and tools to create personalized products with the costs and mass production efficiency.

Neither digital fabrication nor parametric design are new realities; however, it is the integration of both that is creating a new flow of information that allows new effective methodologies for mass customization [42,43,46]. Thus, customization of the products can be achieved through the parameterization of the designs [86], as flexible design structures conceived from the relation between its parameters.

#### 2.4.2. Lightweight Parts

When designing lightweight parts, the goal is to minimize the volume or the amount of material used, without compromising the required mechanical resistance. This means maximizing efficiency in the way the material is used, and it has a series of benefits, both economic and environmental [87,88].

**Figure 2:**
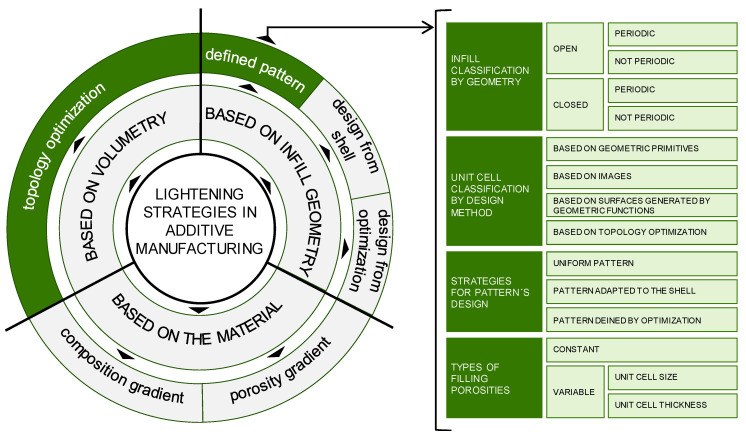
Classification of the lightening strategies of pieces produced by additive manufacturing.

However, by lightening the structures, the geometric complexity of the pieces increases, and that complexity can make it difficult, or even impossible, to manufacture them with traditional technologies. Additive manufacturing addresses this problem by materializing parts layer by layer from digital models and makes these approaches possible. Additive technologies even allow the obtaining of with different materials in a single process [89,90,91]. Many strategies can be adopted to lighten a piece designed to be manufactured with additive technologies, as they present the opportunity to design complex geometries without the restrictions of traditional technologies. Figure 2 presents a scheme with the most relevant ones and indicates with solid green background the ones adopted in the proposed methodology.

## 3. Methodology

The objective of the methodology is to generate lightweight pieces compatible with mass customization strategies and produced by additive manufacturing. The lightening of the pieces is achieved by the topology optimization of the initial volume as well as by the structural optimization of the cellular structures generated for its infill and shell. It is necessary to avoid any intermediate modeling process in which the flow of information is broken, which would act as a frontier for the optimization global process. To reach this goal, the methodology is based on parametric design, where a generative modeling that involves algorithms, structural analysis, and optimization is defined in a continuous workflow. The selection of the most appropriate computational tool according to the particular objectives of the methodology and considering the possible alternatives both for parametric design and optimization process [92] is a key decision. The identification of a suitable software was faced in previous works [41,93], and Grasshopper was the selected tool.

**Figure 3:**
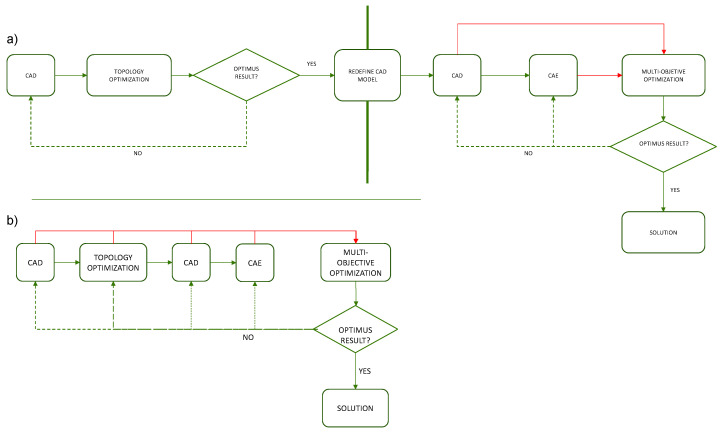
(**a**) Workflow in optimization methodologies of most software. (**b**) Workflow in the optimization methodology proposal in Grasshopper.

Most optimization algorithms stablish workflows from parametric designs, by contrast, in other situations such as topology optimization, in which the variables are unitary and correspond to the discretized elements of the finite element model, it is not possible to establish these workflows with parametric models. Consequently, it is necessary to re-model the parts taking as a reference the results obtained in the topology optimization, this way the data flow between different optimization processes would be broken and therefore the methodology would fail. Figure 3a illustrates this situation.

One of the main advantages of Grasshopper, in the context of the proposed methodology, is that it allows the continuation of modeling the solution through algorithms based on the elements of the mesh resulting from topology optimization. Thanks to this, a continuous workflow can be established between the different algorithms and generative modeling of Grasshopper. This situation is shown in Figure 3b. In this way, any element that intervenes in the process as a variable can be integrated into multi-objective optimization. Thus, the proposed methodology eliminates barriers or stops that could affect the workflow continuity, so the part optimization process, the resulting mesh and the parametric modeling for subsequent optimization are connected through a continuous workflow. Consequently, it is also possible to integrate variables from the algorithms of the topology optimization, or from previous stages, to the subsequent optimizations of the geometry.

Grasshopper [40] is a graphical programming application that uses Rhinoceros (Robert McNeel and Associates, Seattle, WA, USA) [94] interface to visualize the geometries generated by algorithms. As it is an open source software, it benefits from the activity of many researchers who develop algorithms programmed with this language, both in the form of mathematical expressions and programmed sequences. These scripts are shared with other users in the form of plug-ins or add-on. As occurs in any programming language it is unusual to code from scratch, there are always frameworks, plug-ins, and other resources that help coding and include already programmed and grouped sequences for specific functionalities [95]. A review of the most suitable plug-ins considering the utilities of the methodology was carried out in previous works [8,41]. Table 1 shows those selected for this work from the previous works indicated, in which their choice is justified. The proposed plug-ins can be used for the different goals as they introduce frameworks and algorithms already developed that help the methodology scripting. However, it is possible to use any algorithm that the designer considers most suitable for the case of study or even code from zero in any compatible programming language such as Python, C#, NET, or VB.

**Figure 4:**
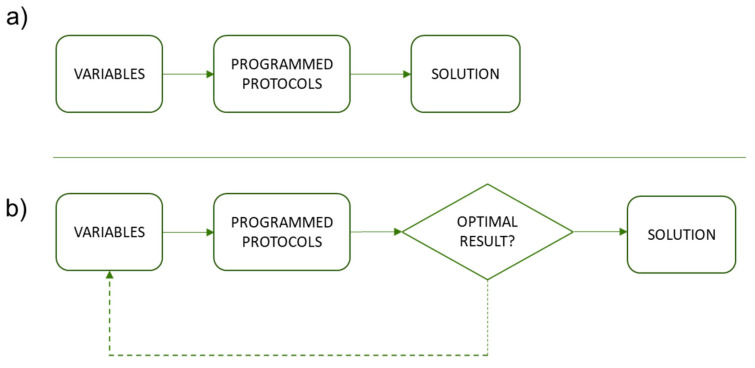
(**a**) Scheme of parametric model with linear sequence. (**b**) Model outline.

The workflow defined through the parametric model of the proposed methodology generates different design solutions without the need to develop new models [96]. This approach is of interest when avoiding the interruption of the workflow of the methodology between the mesh result of the topology optimization and the required parametric design for the further optimizations [97,98]. In this way it is possible to introduce variations in the solution obtained by altering the values of a certain variable without the need to redefine the three-dimensional model. Thus, the programming not only includes linear sequences for the definition of the model, non-linear routines can also be established, as shown in Figure 4.

By means of a parametric design, the final design is not drawn or represented, but calculated. Errors are reduced by eliminating intermediaries and repetitive tasks typical of the traditional design process. Design process becomes more reliable and efficient. Furthermore, a greater variety of solutions are offered automatically, therefore it is possible to choose the most optimal result without entailing additional time or effort [99,100].

**Figure 5:**
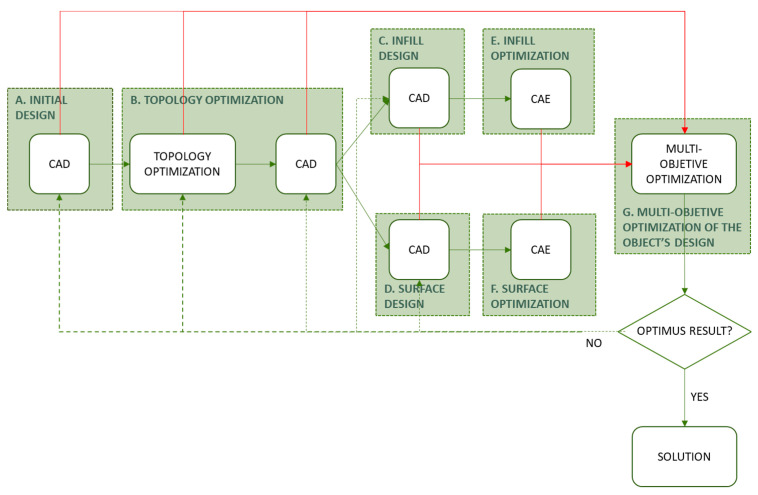
Diagram of the proposed methodology.

This work is focused on additive manufacturing technologies, and from that approach the geometries to be optimized are the general volume, the infill, and the shell of the piece. A diagram of the proposed methodology is shown in Figure 5.

**Figure 6:**
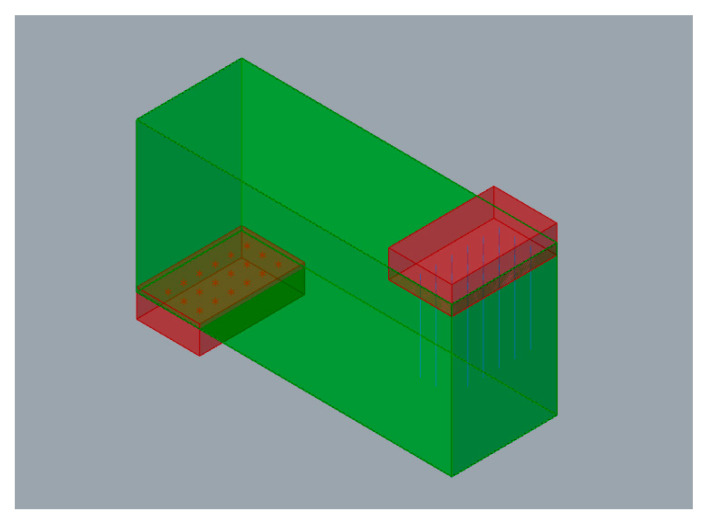
Case study loading conditions.

For clarity, the exposition of the functioning of the methodology is supported by a simple application example, of a cantilever beam of predefined span and variable height and width. Figure 6 schematically represents its loading conditions. Thus, the exposition of each part of the design and optimization problem is accompanied by a representative image of the result obtained for this example.

**Figure 7:**
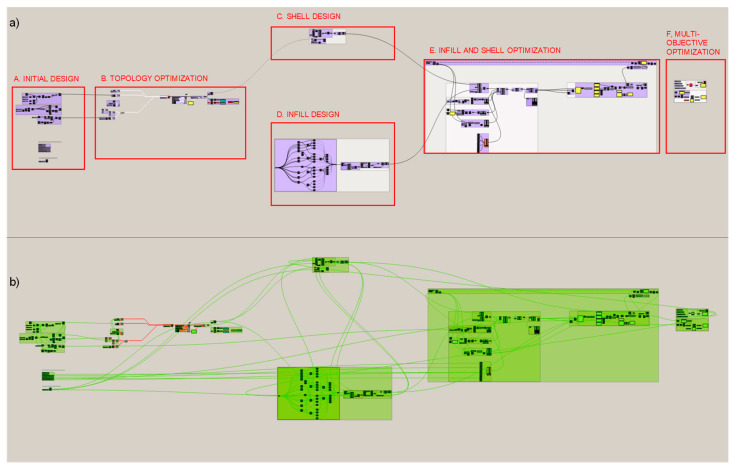
Visual programming structure of the proposed methodology for the case study developed with Grasshopper. (**a**) Structure diagram, (**b**) Data flow in the structure.

Figure 7 shows the proposed methodological structure applied to the cantilever beam case in Grasshopper interface. The same sections as the ones illustrated in Figure 5 are identified. Except for the infill and shell optimization that can be done separately or together in a unitary structure and for the cantilever case study a unique lattice structure for both infill and shell, is defined and optimized. Moreover, in Figure 7 the continuous data flow is represented by green connection lines, where the relations between variables, constants, and functions throughout the methodology is visible. The general programming structure shown describes a continuous workflow through the sequences of operations between the algorithms used for the initial geometry design and the algorithms responsible for the FEA (Finite Element Analysis) and the optimization processes. The parametric design of the piece, as well as any transformation, is defined by sequential actions based on mathematical and geometric programmed functions.

### 3.1. Initial Parametric Design

**Figure 8:**
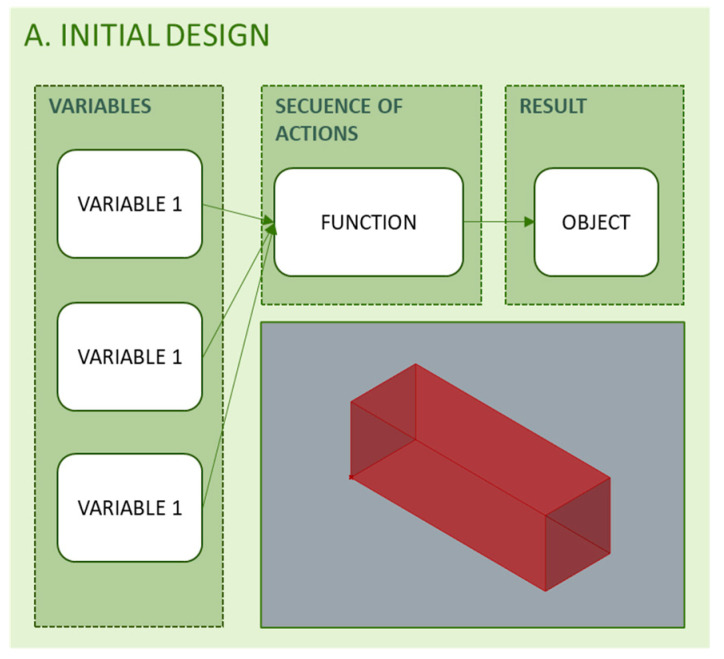
Programming structure for the configuration of the initial volumetric design.

One of the objectives of the methodology is to minimize the influence on the optimized final result from the initial volume, as Figure 8 shows. The initial volume is defined with a parametric design and its variables can be modified to obtain the minimum or maximum value of the objective function to optimize the result. In this sense, the methodology allows the selection of the variables considered initially to be modified at any time, adapting the approach to the particular objective in each case. Thus, as a result, a flexible geometric object is obtained, which is automatically modified when the value of any of its variables is changed.

### 3.2. Topology Optimization

**Figure 9:**
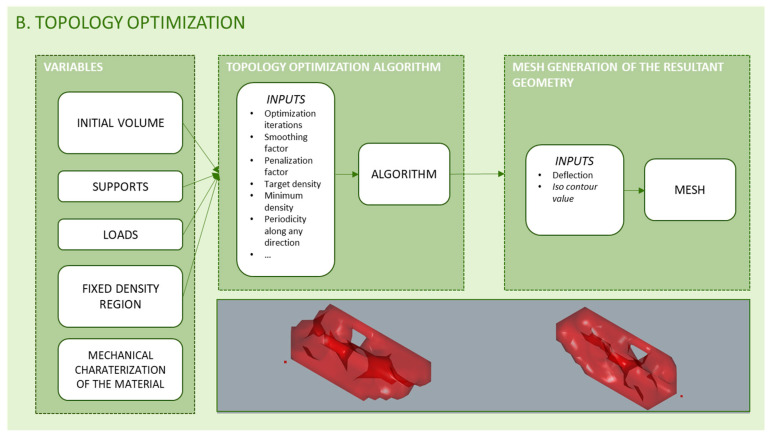
Programming structure for topology optimization.

Topology optimization is performed through a FEA. In this sense, the algorithm inputs are the meshed model to be optimized, the loading conditions, what includes supports and loads, and the mechanical properties of the material, as Figure 9 shows. The programming developed demands resistance values for the material both for the initial topology optimization of the general geometry and for the sizing of the bars of the infill structure. Resistance values provided by filament suppliers cannot be considered, since the manufacturing process and the layered structure generated impose significant changes in the material [21]. Thus, the mechanical characterization of the material required a previous study. In that study, solid specimens were manufactured and tested, since the sizing of the bars of the filling structure considers the resistance of the material with which they are manufactured and the load applied in each area of the piece, not so much the resistance of the cells that define the lattice structure [9].

As previously exposed, although any topology optimization algorithm can be introduced in the methodology, there are several plug-ins available with different algorithm methods such as Solid Isotropic Material Penalization Method (SIMP) in Millipede or Bi-directional Evolutionary Structural Optimization (BESO) in Karamba.

The initial geometry for the optimization process is the result of a sequence of functions with variables from the parametric design of the initial volume. Furthermore, the mesh used to discretize the model using a finite number of elements can be determined with a variable, not a constant. This approach allows the location and the number of nodes where the loads and supports are located to be modified and updated automatically during the process.

The methodology is not limited to the use of a certain topology optimization algorithm. In each case, the most useful alternative can be selected. On the other hand, a topology optimization algorithm will demand a series of input information that can be configured as variables or as constants depending on the problem and the predictable results. Examples of this type of input are, among others, the penalty factor, the maximum volume fraction, the filter’s radius of influence, the number of iterations and manufacturing restrictions. Moreover, in the generation of the resulting meshed geometry there are variables that can be modified to obtain different results, such as the iso contour value.

### 3.3. Shell Design

Additive manufacturing technologies incorporate shell and infill as new aspects to be considered into the design process [8]. When generating the gcode through the laminator software, some variables of these two elements can be configured, but configuration options are limited to standardized presets. Thus, in addition to optimizing the general geometry, the proposed methodology considers it necessary to incorporate the optimization of both aspects, shell, and infill.

**Figure 10:**
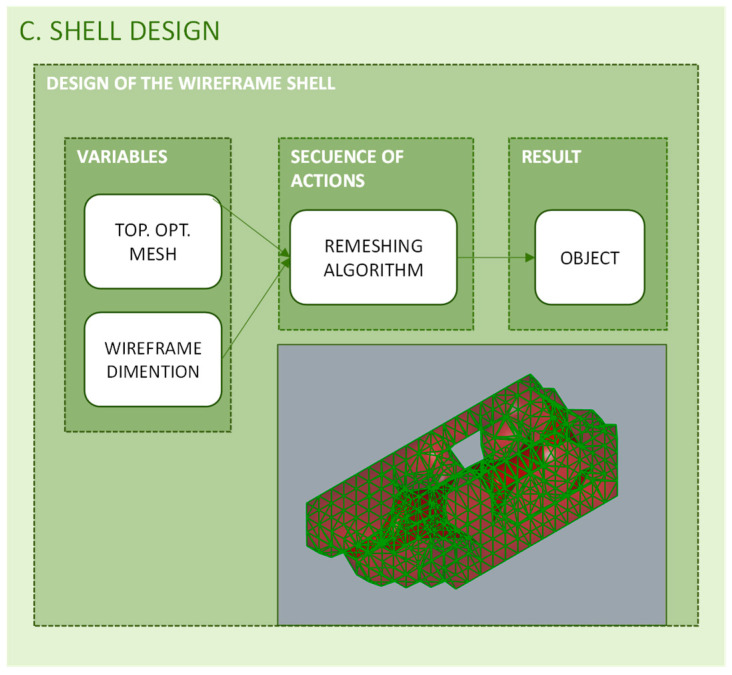
Programming structure for the wireframe shell design.

In that sense, since FDM and SLS (Selective Laser Sintering) were the technologies selected to test the methodology, a porous structure shell was chosen to allow the output of the support material necessary during manufacturing. On the other hand, under the premise of lightening the part without compromising its mechanical behavior, a wireframe structure with variable cross sections and working collaboratively with the infill structure has a better mechanical behavior than a continuous closed shell [101]. In Figure 10 the programming structure used for shell design is shown.

### 3.4. Infill Design

Infill patterns and percentages are offered in slicing software as predetermined options. The objective of the proposed methodology is to optimize not only the boundary geometry of the piece, but also the shell and infill that conforms it. To achieve this goal, the predetermined options are not valid, and the infill pattern must be designed from Grasshopper software in a continuous workflow.

**Figure 11:**
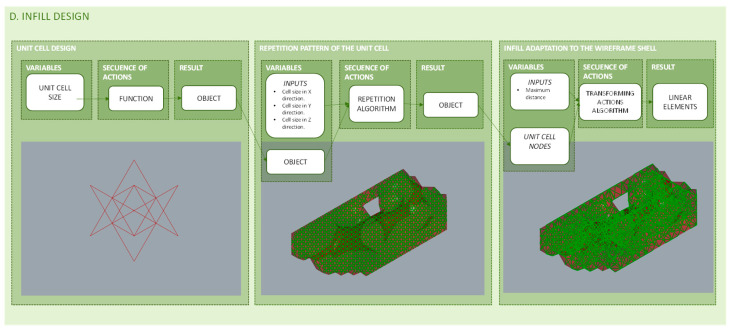
Programming structure for infill design and for its adaptation to the lightweight shell structure.

Although any infill design is supported by the methodology, to be more specific, periodic opened structures are chosen. These structures have shown to be stronger than non-periodic [102]. Moreover, open structures allow the reduction of the amount of material used while maintaining its rigidity and strength [103]. The diagram in Figure 11 summarizes the programming structure detailed below for the generation of the infill pattern design. As can be seen, the diagram distinguishes three blocks that include in each case the variables considered, the sequence of actions that takes place and the results obtained. The first block deals with the design of the unit cell. The second develops the programed sequence where the unit cell is repeated to form larger structures filling the topologically optimized geometry. In addition, the third is oriented to the adaptation of the resulting infill structure when meeting the wireframe shell that defines the geometry of the piece. This last block welds the endpoints from the extremes of the infill structure in the contour of the geometry with the nearest nodes of the shell structure.

#### 3.4.1. Unit Cell Design

The proposed methodology includes the parametric design of the infill unit cell. A revision on cellular structures is made in previous works and the selection of lattice structures is justified [41,104].

#### 3.4.2. Cell Repetition Pattern Unit

On the other hand, the proposed methodology allows different repetition patterns of the infill‘s unit cell depending on the geometry of the piece. In the case of a predominantly flat volume, it would be convenient to make a conformal lattice structure. However, in any other case, it would be convenient to perform a direct repeating pattern with an orthogonal axis orientation configuring a uniform lattice structure. Depending on the case, one algorithm or another will be used, both are available in the methodology proposed.

#### 3.4.3. Infill and Shell Coherence

A wireframe structure is designed for both infill and shell; however they are independently constructed, therefore it is necessary to ensure that nodes from the infill and the wireframe shell are weld into a coherent structure. This way, the lattice structure, formed by the union of the shell and infill structures, works in a unitary way and the stresses of the bars of the wireframe shell are transmitted to the bars of the infill structure.

**Figure 12:**
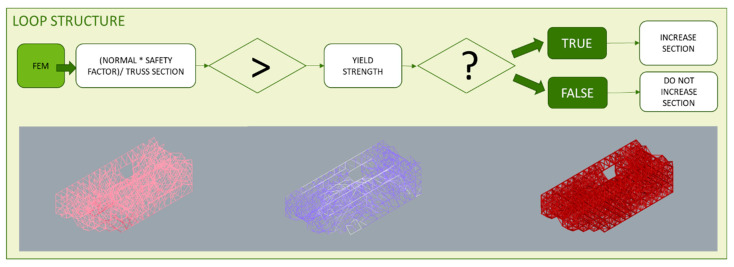
Loop sequence for the cross-section assignment.

The lattice structure previously designed is now optimized based on a FEA of the wired elements. A size optimization is developed and, at first, the minimum section feasible for the additive manufacturing technology chosen is assigned to each element. For this minimum section, normal stresses are calculated for the load case and material assigned. Each normal value is compared one by one with the elastic limit of the material taking into account a safety coefficient, if the normal value exceeds it, the bar section is increased. However, if it resists, even complying with the safety factor, the section of the bar is maintained. Both the FEA and the described sequence are repeated until no bar exceeds the elastic limit of the material. These iterations are performed through the loop programming structure shown in the diagram of Figure 12, where a comparison and a conditional operator included as well as functions that perform the specific actions.

**Figure 13:**
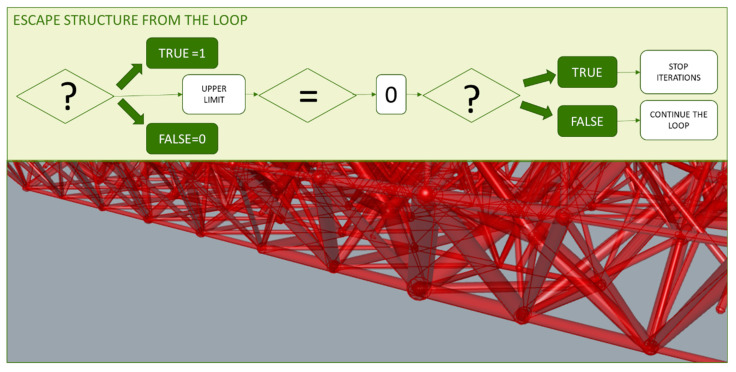
Programming structure of the escape sequence of the previous loop.

On the other hand, the loop escape sequence is configured from the result of a conditional structure, where integer values are assigned to the resultant Boolean values and its upper limit of compared to the value 0 that corresponds to the negative value. This way, if there is not any value that exceeds the elastic limit mentioned before, the iterations of the loop will stop and no more FEA of the lattice structure nor further sections assignations will be done. Consequently, a heterogeneous porous structure is generated, as shown in Figure 13.

### 3.5. Multi-Objective Optimization Problem

**Figure 14:**
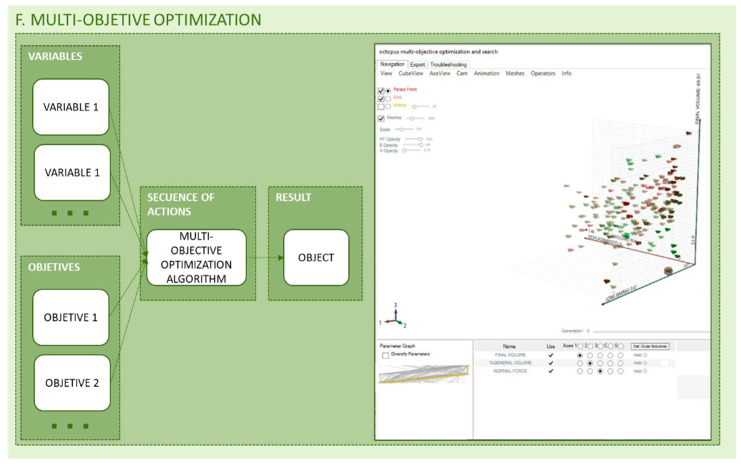
Programming structure of multi-objective optimization.

The main objective of the methodology proposed, and therefore of the optimization problems involved, is to generate a lightweight piece from both the general volume and the material to be used while ensuring a good mechanical behavior. To achieve this goal more than one objective must be set and therefore a multi-objective problem is programmed. For the beam example Octopus plug-in was used, the multi-objective optimization algorithms inside the plug-in are based on SPEA-2 and HypE algorithm from ETH Zurich (Eidgenössische Technische Hochschule Zürich, Zurich, Switzerland). Iterations of the entire design as well as its optimization processes, such as topology optimization or lattice structure optimization of the piece, are performed. For each iteration different values, previously defined by the designer as viable values, are assigned to the variables of the parametric design to generate different viable solutions. This way, different populations of solutions are generated and recorded to guide the designer in the search and decision of the best solution for its manufacture. The interface of the plug-in Octopus allows the designer to compare the Pareto solutions looking at their objective values and the variables that originate them, to choose the most suitable final solution ready to export in a compatible format to manufacture. Figure 14 illustrates a scheme of the multi-objective optimization problem and Octopus interface. As in the case of topology optimization, the methodology allows the selection of the most appropriate method to be applied to each problem. In any case, for the application in the proposed methodology, optimization algorithms based on metaheuristic methods are more interesting since they condition the final solution to a lesser extent and explore the search space more efficiently.

The general parameters considered for the multi-objective optimization problem of the proposed methodology applied to the beam example are the ones showed in Table 2. However, the proposed methodology can take any variable from the parametric design, not only of the initial volume before the topology optimization, but also from the infill or shell design. This way, the designer fixes the constraints and limitations for the optimization problem from the range of values of the variables for the parametric design defined for the specific case. One of the mayor limitations in additive technologies is the minimum strut diameter that can be manufactured, for this reason, the minimum strut diameter is stablished as a constant, to be defined by the designer for the specific technology to be used. Not all limitations are initially introduced this way, such as overhanging structures, as the methodology can be applied to other additive technologies that do not require additional support material. However, maximum strut length is restricted to the range of values assigned to the variables of the wireframe shell size and the lattice infill size introduced in the multi-objective optimization problem and it is defined by the designer from the technology’s characteristics.

First, the main objective, as mentioned before, is to minimize the amount of material without compromising the resistance of the piece, therefore it is necessary to establish the objectives within the optimization problem, which can be configured from different mechanical concepts, such as minimizing displacement or energy deformation, maximize stiffness, etc. However, it is possible to introduce as many objectives as deemed necessary depending on the specific case.

Secondly, any variable introduced in the parametric design as well as in the optimization problems, such as topology optimization or size optimization of the lattice structure, can be considered in the multi-objective optimization problem. For the example given of the beam, the location of the loads and supports that determines the geometry that the topology optimization acquires is considered, as well as the geometric variables that determine the initial volume of the piece or the final lattice structure.

Finally, the restrictions of the described variables are determined from the range of admissible values of the variables. In addition, mathematical relationships are established between the different variables to restrict the search for solutions to the feasible region.

## 4. Results and Discussion

The proposed methodology, described in the previous section, integrates structural optimization such as topology and size optimization as well as multi-objective optimization through a continuous workflow developed with Grasshopper, a graphic coding application integrated in Rhinoceros. From these methodological bases, two possibilities arise:

(1) Methodology with a single multi-objective optimization algorithm

(2) Hierarchical methodology with more than one multi-objective optimization algorithm

To illustrate the approach and operation of the proposed methodologies, highly simplified schematics are presented to represent the programmed structure of the continuous data flow throughout the optimization and design problem. The methodology proposes the use of scripts or plug-ins with developed algorithms for specific tasks such as optimization problems or structural analysis acting as frameworks inside Grasshopper’s language. However, Grasshopper supports other programming language to import or write other algorithms. This flexibility allows the methodology to maintain updated as well as to easily adapt to different case studies while maintaining the programming structure and elements.

**Figure 15:**
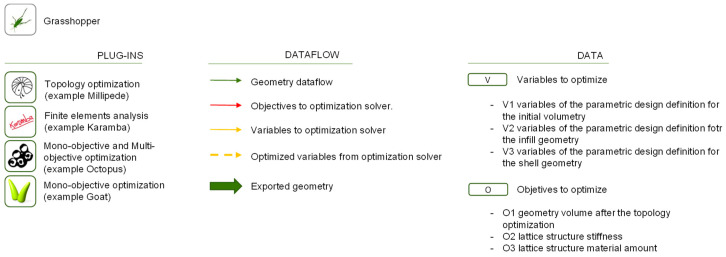
Legend of the symbols used in the diagrams of the different methodologies.

Optimization and FEA algorithms are pointed out in the methodology diagrams and are represented by its plug-in logo images for a more intuitive understanding. The letter V represents the variables that act as inputs to the algorithms that generate the geometry, and the O represents the objectives extracted from the geometry data or from the FEA solver data. On the other hand, the cubes, represent the geometric objects generated from Grasshopper using mathematical relationships or algorithms. The lines and arrows represent the flow of information through the functions and algorithms of the methodology. The type of data flow is represented in different colors further explained in the Figure 15.

Depending on the ubication of the multi-objective algorithms in the workflow two different methodological approaches are presented below; continuous and hierarchical.

### 4.1. Continuous Methodology

**Figure 16:**
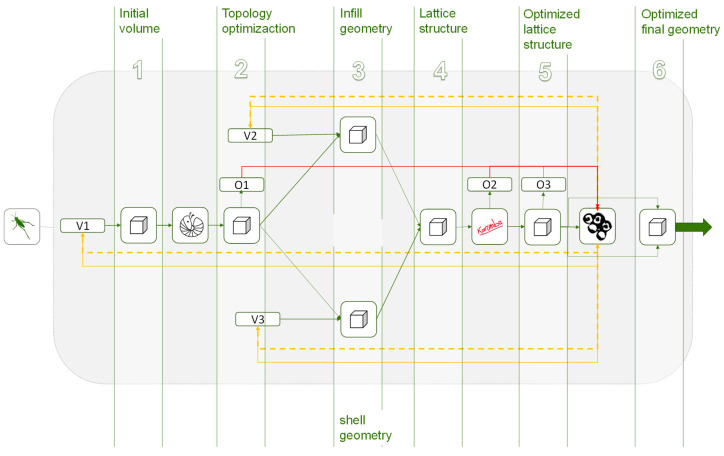
Methodology using a single-optimization solver.

As can be seen in the diagram in Figure 16, the geometry is generated parametrically from initial variables. A topology optimization is then carried out from the resultant geometry and the resultant mesh is analyzed through a succession of algorithms and functions in order to generate from it, on one hand, the lattice infill and, on the other, the wireframe shell. Both structures are weld together into a single coherent structure which is optimized with a multi-objective algorithm at the end to compare and select the more suitable optimized solution in each case.

### 4.2. Hierarchical Methodology

Octopus multi-objective optimization algorithms produce solutions from the random search of the variables making iterations over the entire structure of the programming. The more variables and objectives introduced in a single problem, the more iterations will be necessary to select the best genome or solution, which implies a greater consumption of computing resources.

On the other hand, depending on the infill geometry and its relation to the contour shell, it may be interesting to break the optimization problem into subproblems to have greater control over the optimization of each part.

**Figure 17:**
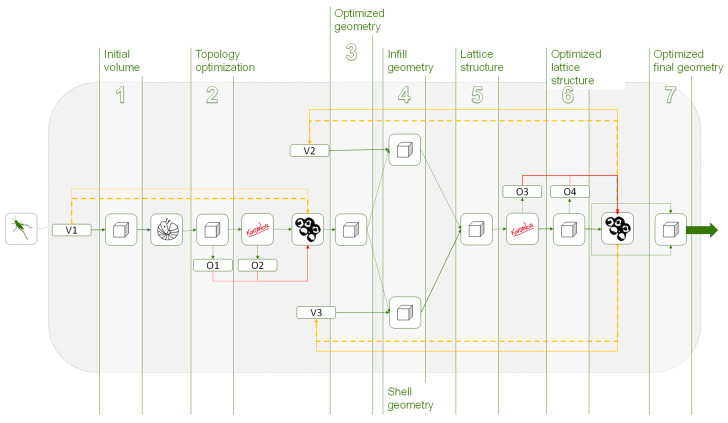
Hierarchical methodology with more than one multi-objective optimization algorithm in which the optimization problem is decomposed into 2; initial volume and lattices structure that includes the infill and the shell.

**Figure 18:**
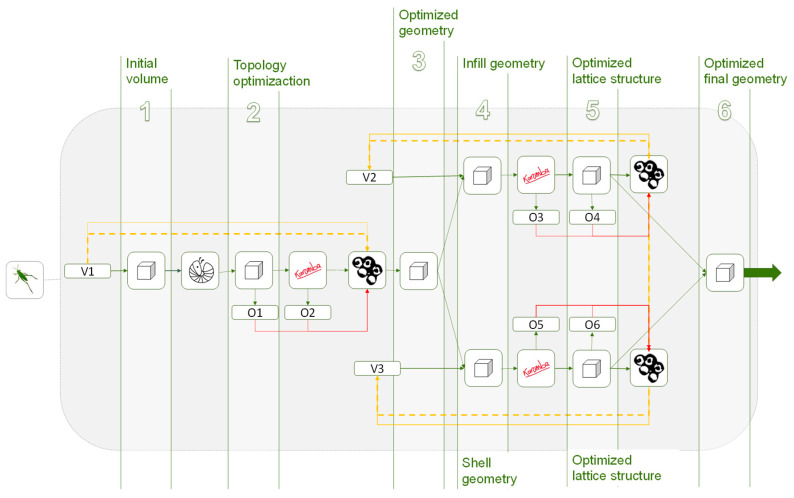
Hierarchical methodology with more than one multi-objective optimization algorithm in which the optimization problem is decomposed into 3; initial volume, infill structure, and wireframe shell.

In response to these situations, a hierarchical methodology is proposed, in which the general volume is optimized several times in different stages of the problem. As shown in Figure 17 and Figure 18 a first multi-objective optimization is done to the general volume after the topology optimization, and a second optimization is carried out to the cellular infill and shell structures, either jointly or independently.

As represented in Figure 17, after the topology optimization, the optimization problem is separated into 2 multi-objective optimization subproblems. This proposed hierarchical methodology, in which the problem is broken down into subproblems, allows the problem to be decomposed as much as necessary while there is a flow of data throughout the problem.

**Figure 19:**
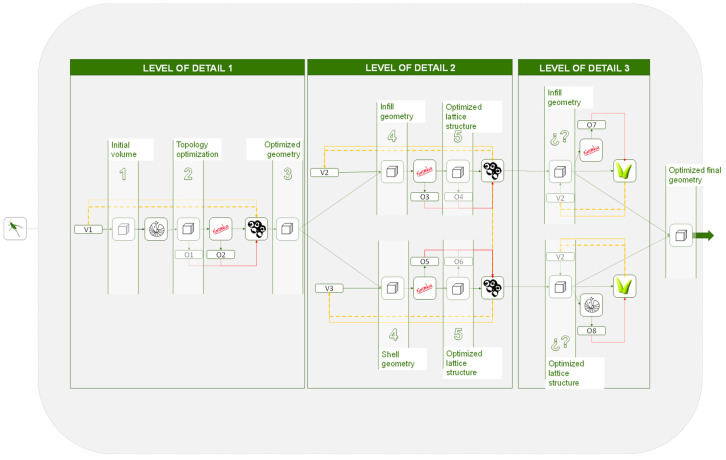
Hierarchical methodology applied to different levels of detail.

**Figure 20:**
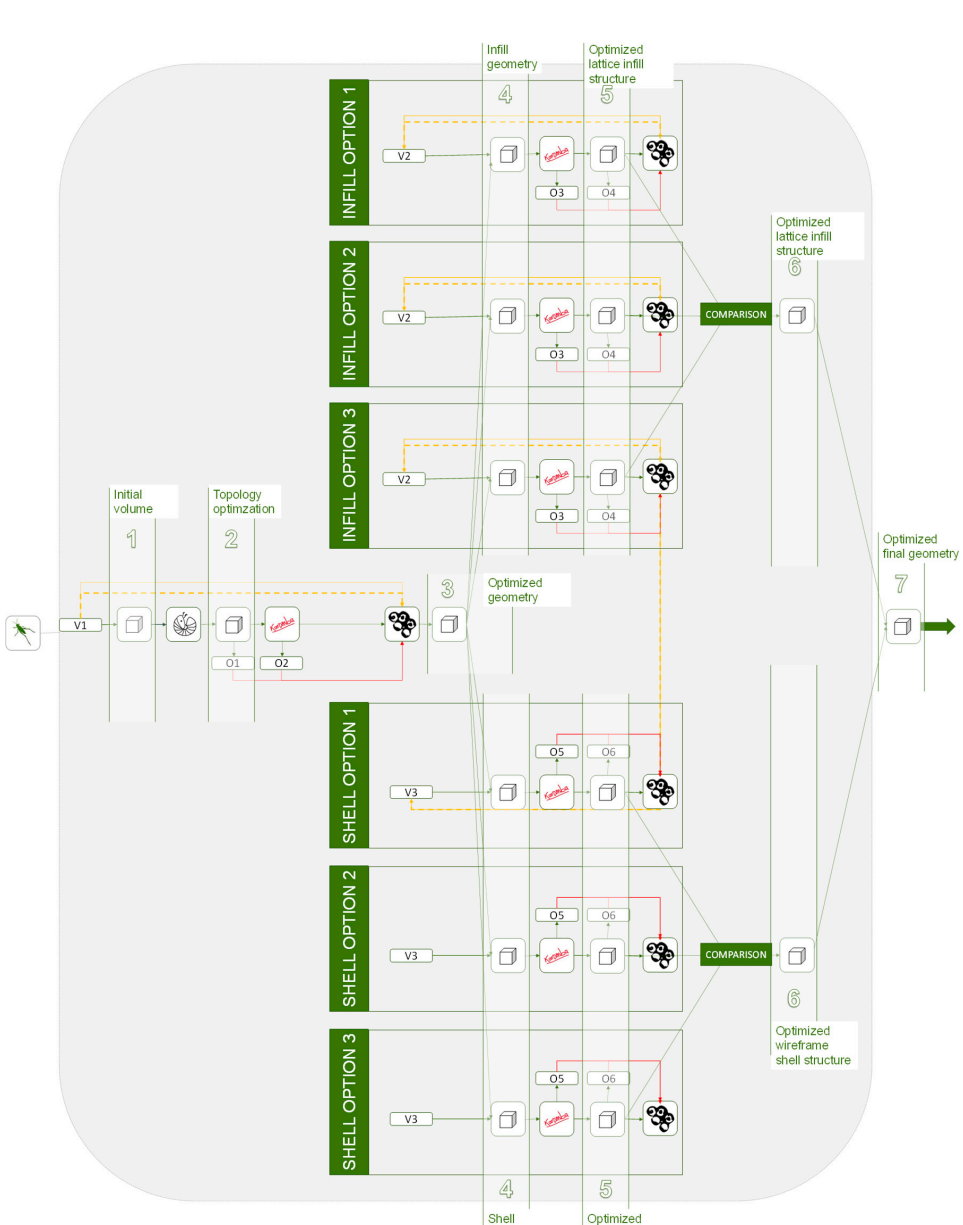
Hierarchical methodology applied to design alternatives.

It is possible to decide which methodology is more interesting and efficient in each case once much of the optimization problem has been modeled by including the optimization algorithm in the design stage of interest. On one hand the continuous methodology is preferred when there are few variables to be taken into account in order to be able to search through a representative number of solutions with a viable consumption of computing resources. On the other hand, the hierarchical methodology with more than one optimization solver is of special interest in the following cases:

(1) High degree of detail: In those cases, in which a high degree of detail needed without initially conditioning the final geometry, the hierarchical methodology is the most suitable. By subdividing the problem in different levels of detail, the geometry is optimized from the general volume to the finest detail, making decisions throughout the process and having absolute control over the final result. In the diagram of Figure 19 there are only 3 degrees of detail; however, the problem can be decomposed to further details or even to its different geometry parts. In addition, other solvers or algorithms can be incorporated at any point in the optimization process and included within the methodology. For instance, in Figure 19 an example of one possible application is illustrated and Goat, mono-objective optimization solver is introduced to optimize in further degree of detail.

(2) Design alternatives: When it is interesting to assess different design alternatives to choose the most effective the hierarchical methodology is the most suitable option. Once the problem is defined, it is easy to branch any subproblem into different design alternatives at any point of the process. Figure 20 illustrates a diagram of an example with the explained possibilities.

**Figure 21:**
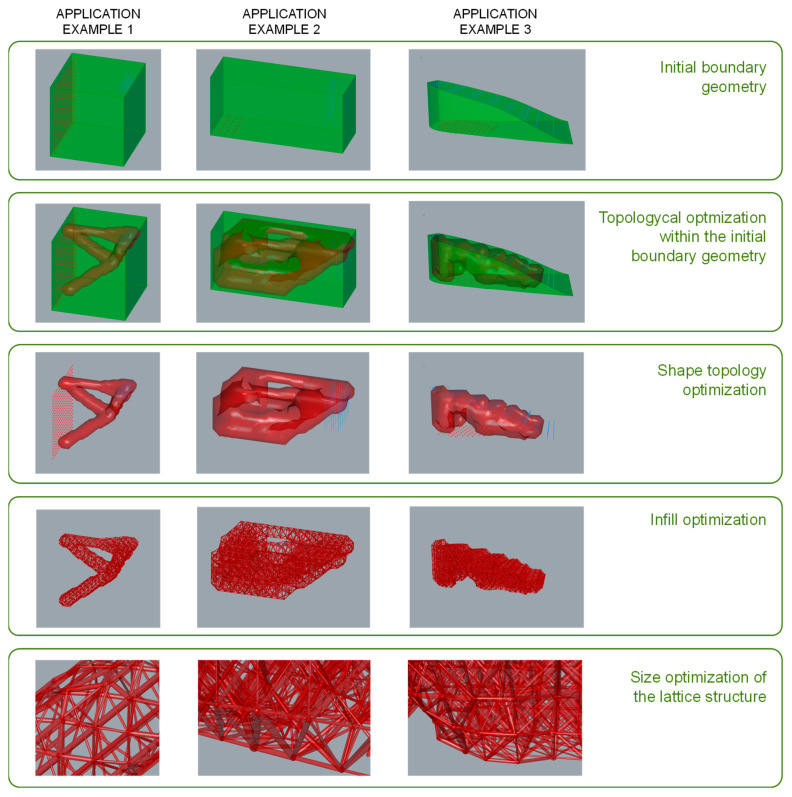
Methodology application examples.

The methodology proposed in this work has been applied to different case studies to validate its operation. Its application in the design of a beam has been used as an example in some sections of this work to support specific parts of the exposition and to illustrate how the methodology contributes to the definition of the final solution through its different stages. Figure 21 illustrates the application of the methodology to the beam and two other examples: a wall hook and the heel of a shoe. The main stages and aspects of the design and optimization process are outlined and represented.

## 5. Conclusions

The proposed methodology successfully integrates three technologies whose potential and utility are significantly increased when they work together, taking advantage of their synergies. The results obtained are considered satisfactory and represent optimized design solutions that improve those that would be achieved using any of the technologies considered independently.

The methodological approach presented in this work integrates topology optimization with mono-objective and multi-objective optimization in a continuous workflow using parametric design. This general achievement allows the application of the methodology to different fields and represents a useful design and optimization tool for additive manufacturing scenarios. It allows the taking of advantage of the greater geometric freedom allowed by additive manufacturing, and to explore the possibilities of these manufacturing technologies.

The adaptation of the designs to individual needs and preferences that is allowed by the methodology, makes this approach of great interest for mass customization strategies. In this sense, the methodology can be understood as a design resource for this kind of contexts, with great application opportunities in fields such as medicine, fashion design, or disciplines such as ergonomics.

In addition, the methodology allows the level of flexibility required in each case, the design problem can be decomposed into hierarchical optimization subproblems for greater control by the designer, and without any barrier in the data flow or in the design process.

On the other hand, and from teaching approaches, this methodology is expected to be one of the main resources to be used in the framework of the 2020 Call for Teaching Innovation Projects requested by the authors as members of the Teaching Innovation Group (GID2016-28) of the UNED. The authors want to identify key criteria and aspects of influence on the success of additive manufacturing process carried out by the students, in order to incorporate them within a multicriteria hierarchical structure able to support decision-making for layout design and equipment future acquisitions. Since the developed methodology integrates and explores the synergies between three key technologies, such as additive manufacturing, parametric design, and optimization processes, it can guide the identification of the key criteria and aspects of influence, and its role in this teaching initiative is expected to be significant, both for researchers and students.

Finally, the results obtained considering FDM 3D printers as the manufacturing technologies for this work, encourage the authors to launch similar experiences to validate and improve the application of the methodology with other additive technologies. On the other hand, the authors are currently in the initial stages of studies focused on the analysis of the mechanical behavior of specific designs obtained from applying the methodology. It is expected that the results obtained will serve to improve the functioning of the methodology, validating the results of the simulations ran from the mechanical characterization of the material faced in previous works.

## Figures and Tables

**Table 1:** Classification of plug-ins considered to be included in the methodology.

Structural Analysis Based in FEM	Topology Optimization	Mono-Objective Optimization	Multi-Objective Optimization	Cellular Infill	Loops
Karamba Millipede	Toppot (2d) Millipide (2d & 3d) Topos Mololith	Galapagos Goat Millipede (structural optimization) Karamba (structural optimization) Octopus	Octopus Octopus e.	Monolith Crystallon Intralattice	Anemone Loop Octopus loop Hoopsnake

**Table 2:** Multi-objective optimization parameters applied to the beam example.

	Objectives	Variables	Restrictions
**General volume**	Minimum volume	Load position Initial geometry variables	Distance and direction restrictions with the initial geometry given by the load cases Geometry restrictions
**Shell**	Maximum stiffness Minimum volume	Wireframe shell bar section Wireframe shell size	Minimum and maximum wireframe shell bar section Geometry restrictions
**Infill**	Maximum stiffness Minimum volume	Infill bar section Lattice infill size	Minimum and maximum Infill bar section Geometry restrictions

## References

[1] Gibson I., Rosen D.W., Stucker B. (2010). Additive Manufacturing Technologies. Rapid Prototyping o Direct Digital Manufacturing.

[2] International Organization for Standardization (2015). ISO 17296-2:2015, Additive Manufacturing General Principles. Part 2: Overview of Process Categories and Raw Materials.

[3] Tofail S.A.M., Koumoulos E.P., Bandyopadhyay A., Bose S., O’Donoghue L., Charitidis C. (2018). Additive manufacturing: Scientific and technological challenges, market uptake and opportunities. Mater. Today.

[4] Pérez-Pérez M., Gómez E., Sebastián M. (2018). Delphi prospection on additive manufacturing in 2030: Implications for education and employment in Spain. Materials.

[5] Bikas H., Stavropoulos P., Chryssolouris G. (2016). Additive manufacturing methods and modelling approaches: A critical review. Int. J. Adv. Manuf. Technol..

[6] Abdulhameed O., Al-Ahmari A., Ameen W., Mian S.H. (2019). Additive manufacturing: Challenges, trends, and applications. Adv. Mech. Eng..

[7] Attaran M. (2017). The rise of 3-D printing: The advantages of additive manufacturing over traditional manufacturing. Bus. Horiz..

[8] Garcia-Dominguez A., Claver-Gil J., Sebastian-Perez M.A. (2018). Propuestas para la optimización de piezas para fabricación aditiva. Dyna Ing. E Ind..

[9] García-Domínguez A., Claver J., Camacho A.M., Sebastián M.A. (2019). Considerations on the applicability of test methods for mechanical characterization of materials manufactured by FDM. Materials.

[10] Rodríguez-Panes A., Claver J., Camacho A.M. (2018). The influence of manufacturing parameters on the mechanical behaviour of PLA and ABS pieces manufactured by FDM: A comparative analysis. Materials.

[11] Zaldivar R.J., Witkin D.B., McLouth T., Patel D.N., Schmitt K., Nokes J.P. (2017). Influence of processing and orientation print effects on the mechanical and thermal behavior of 3D-printed ULTEM^®^ 9085 material. Addit. Manuf..

[12] Wang X., Zhao L., Fuh J.Y.H., Lee H.P. (2019). Effect of porosity on mechanical properties of 3D printed polymers: Experiments and micromechanical modeling based on *x*-ray computed tomography analysis. Polymers.

[13] Popescu D., Zapciu A., Amza C., Baciu F., Marinescu R. (2018). FDM process parameters influence over the mechanical properties of polymer specimens: A review. Polym. Test..

[14] Wang L., Gramlich W.M., Gardner D.J. (2017). Improving the impact strength of poly(lactic acid) (PLA) in fused layer modeling (FLM). Polymer.

[15] Bajerski P., Pęcherski R.B. (2017). Influence of additive manufacturing technology on mechanical properties of glass-filled fine polyamide PA3200GF. Eng. Trans..

[16] Mehraein H. (2018). Impact of Process Parameters on Mechanical Properties of 3D Printed Polycaprolactone Parts. Master’s Thesis.

[17] Aw Y.Y., Yeoh C.K., Idris M.A., Teh P.L., Hamzah K.A., Sazali S.A. (2018). Effect of Printing parameters on tensile, dynamic mechanical, and thermoelectric properties of FDM 3D printed CABS/ZnO composites. Materials.

[18] Ćwikła G., Grabowik C., Kalinowski K., Paprocka I., Ociepka P. (2017). The influence of printing parameters on selected mechanical properties of FDM/FFF 3D-printed parts. IOP Conf. Ser. Mater. Sci. Eng..

[19] Samykano M., Selvamani S.K., Kadirgama K., Ngui W.K., Kanagaraj G., Sudhakar K. (2019). Mechanical property of FDM printed ABS: Influence of printing parameters. Int. J. Adv. Manuf. Technol..

[20] García Plaza E., Núñez López P., Caminero Torija M., Chacón Muñoz J. (2019). Analysis of PLA geometric properties processed by FFF additive manufacturing: Effects of process parameters and plate-extruder precision motion. Polymers.

[21] Goh G.D., Yap Y.L., Tan H.K.J., Sing S.L., Goh G.L., Yeong W.Y. (2020). Process–structure–properties in polymer additive manufacturing via material extrusion: A review. Crit. Rev. Solid State Mater. Sci..

[22] Valerga A.P., Batista M., Fernandez-Vidal S., Gamez A. (2019). Impact of chemical post-processing in fused deposition modelling (FDM) on polylactic acid (PLA) surface quality and structure. Polymers.

[23] Valerga A.P., Batista M., Salguero J., Girot F. (2018). Influence of PLA Filament Conditions on Characteristics of FDM Parts. Materials.

[24] Yin J., Lu C., Fu J., Huang Y., Zheng Y. (2018). Interfacial bonding during multi-material fused deposition modeling (FDM) process due to inter-molecular diffusion. Mater. Des..

[25] Dizon J.R.C., Espera A.H., Chen Q., Advincula R.C. (2018). Mechanical characterization of 3D-printed polymers. Addit. Manuf..

[26] Singh R., Kumar R., Farina I., Colangelo F., Feo L., Fraternali F. (2019). Multi-material additive manufacturing of sustainable innovative materials and structures. Polymers.

[27] Striemann P., Hülsbusch D., Niedermeier M., Walther F. (2020). Optimization and quality evaluation of the interlayer bonding performance of additively manufactured polymer structures. Polymers.

[28] Garcia-Dominguez A., Claver J., Camacho A.M., Sebastian M.A. (2020). Analysis of general and specific standardization developments in additive manufacturing from a materials and technological approach. IEEE Access.

[29] Forster A.M. (2015). Materials Testing Standards for Additive Manufacturing of Polymer Materials: State of the Art and Standards Applicability.

[30] Lubombo C., Huneault M.A. (2018). Effect of infill patterns on the mechanical performance of lightweight 3D-printed cellular PLA parts. Mater. Today Commun..

[31] Chacón J.M., Caminero M.A., Núñez P.J., García-Plaza E., García-Moreno I., Reverte J.M. (2019). Additive manufacturing of continuous fibre reinforced thermoplastic composites using fused deposition modelling: Effect of process parameters on mechanical properties. Compos. Sci. Technol..

[32] Akhoundi B., Behravesh A.H. (2019). Effect of filling pattern on the tensile and flexural mechanical properties of FDM 3D printed products. Exp. Mech..

[33] Fernandez-Vicente M., Calle W., Ferrandiz S., Conejero A. (2016). Effect of infill parameters on tensile mechanical behavior in desktop 3D printing. 3D Print. Addit. Manuf..

[34] Zanetti E.M., Aldieri A., Terzini M., Calì M., Franceschini G., Bignardi C. (2017). Additively manufactured custom load-bearing implantable devices: Grounds for caution What this review adds. Australas. Med. J..

[35] Ambu R., Motta A., Cali M. (2020). Design of a customized neck orthosis for FDM manufacturing with a new sustainable bio-composite. Design Tools and Methods in Industrial Engineering.

[36] Leary M. (2019). Design for Additive Manufacturing.

[37] Zhang Y., Bernard A., Gupta R.K., Harik R. (2014). Evaluating the design for additive manufacturing: A process planning perspective. Procedia CIRP.

[38] Medellin-Castillo H.I., Zaragoza-Siqueiros J. (2019). Design and manufacturing strategies for fused deposition modelling in additive manufacturing: A review. Chinese J. Mech. Eng..

[39] Huang J., Chen Q., Jiang H., Zou B., Li L., Liu J., Yu H. (2020). A survey of design methods for material extrusion polymer 3D printing. Virtual Phys. Prototyp..

[40] Grasshopper-Algorithmic Modeling for Rhino. https://www.grasshopper3d.com/.

[41] García-Domínguez A. (2019). Methodology for the Optimization of Parts Obtained by Additive Manufacturing into Mass Customization Strategies. Ph.D. Thesis.

[42] Van Stralen M. Mass Customization: A critical perspective on parametric design, digital fabrication and design democratization. Proceedings of the 22th Conference of the Iberoamerican Society of Digital Graphics.

[43] Radder L., Louw L. (1999). Mass customization and mass production. TQM Mag..

[44] Tsigkas A., Chatzopoulos C. From design to manufacturing for mass customization. Proceedings of the 3rd International Conference MCP.

[45] Smith S., Jiao R., Chu C.H. (2013). Editorial: Advances in mass customization. J. Intell. Manuf..

[46] Paoletti I. (2017). Mass customization with additive manufacturing: New perspectives for multi performative building components in architecture. Procedia Eng..

[47] García-Domínguez A., Claver J., Sebastián M.A. Mass customasing through designs parametrisation. Proceedings of the 22nd International Conference on Project Management and Engineering.

[48] Teng C.-L., Chen J.-Y., Chang T.-L., Hsiao S.-K., Hsieh Y.-K., Villalobos Gorday K., Cheng Y.-L., Wang J. (2020). Design of photocurable, biodegradable scaffolds for liver lobule regeneration via digital light process-additive manufacturing. Biofabrication.

[49] Griffin M., Castro N., Bas O., Saifzadeh S., Butler P., Hutmacher D.W. (2020). The current versatility of polyurethane three-dimensional printing for biomedical applications. Tissue Eng. Part B Rev..

[50] Sherwood R.G., Murphy N., Kearns G., Barry C. (2020). The use of 3D printing technology in the creation of patient-specific facial prostheses. Irish J. Med. Sci..

[51] Culmone C., Henselmans P.W.J., van Starkenburg R.I.B., Breedveld P. (2020). Exploring non-assembly 3D printing for novel compliant surgical devices. PLoS ONE.

[52] Tan Y.J.N., Yong W.P., Kochhar J.S., Khanolkar J., Yao X., Sun Y., Ao C.K., Soh S. (2020). On-demand fully customizable drug tablets via 3D printing technology for personalized medicine. J. Control. Release.

[53] Chen G., Xu Y., Philip Chi Lip K.C.L., Kang L. (2020). Pharmaceutical applications of 3D printing. Addit. Manuf..

[54] Mohammed A., Elshaer A., Sareh P., Elsayed M., Hassanin H. (2020). Additive manufacturing technologies for drug delivery applications. Int. J. Pharm..

[55] Dong X.-P., Zhang Y.-W., Pei Y.-J., Wang Z., Zhang X.-X., Yu X.-L., Ai Z.-Z., Mei Y.-X., Li J.-N. (2020). Three-dimensional printing for the accurate orthopedics: Clinical cases analysis. Bio-Design Manuf..

[56] Javaid M., Haleem A. (2020). 3D printed tissue and organ using additive manufacturing: An overview. Clin. Epidemiol. Glob. Heal..

[57] Paolini A., Kollmannsberger S., Rank E. (2019). Additive manufacturing in construction: A review on processes, applications, and digital planning methods. Addit. Manuf..

[58] Johnson K., Zemba M., Conner B.P., Walker J., Burden E., Rogers K., Cwiok K.R., Macdonald E., Cortes P. (2019). Digital manufacturing of pathologically-complex 3D printed antennas. IEEE Access.

[59] Lee J.-Y., An J., Chua C.K. (2017). Fundamentals and applications of 3D printing for novel materials. Appl. Mater. Today.

[60] Van de Werken N., Tekinalp H., Khanbolouki P., Ozcan S., Williams A., Tehrani M. (2020). Additively manufactured carbon fiber-reinforced composites: State of the art and perspective. Addit. Manuf..

[61] Rodriguez-Prieto A., Camacho A.M., Aragon A.M., Sebastian M.A., Yanguas-Gil A. (2018). Polymers selection for harsh environments to be processed using additive manufacturing techniques. IEEE Access.

[62] Culot G., Orzes G., Sartor M., Nassimbeni G. (2020). The future of manufacturing: A delphi-based scenario analysis on industry 4.0. Technol. Forecast. Soc. Chang..

[63] Elhoone H., Zhang T., Anwar M., Desai S. (2019). Cyber-based design for additive manufacturing using artificial neural networks for industry 4.0. Int. J. Prod. Res..

[64] Haleem A., Javaid M. (2019). Additive manufacturing applications in industry 4.0: A review. J. Ind. Integr. Manag..

[65] Ceruti A., Marzocca P., Liverani A., Bil C. (2019). Maintenance in aeronautics in an industry 4.0 context: The role of augmented reality and additive manufacturing. J. Comput. Des. Eng..

[66] Mehrpouya M., Dehghanghadikolaei A., Fotovvati B., Vosooghnia A., Emamian S.S., Gisario A. (2019). The potential of additive manufacturing in the smart factory industrial 4.0: A review. Appl. Sci..

[67] Caetano I., Santos L., Leitão A. (2020). Computational design in architecture: Defining parametric, generative, and algorithmic design. Front. Archit. Res..

[68] Kalay Y.E. (1989). Modelling Objects and Environments (Principles of Computer Aided Design).

[69] Janssen P., Stouffs R. Types of parametric modelling. Proceedings of the 20th International Conference of the Association Computer-Aided Architectural Design Research in Asia (CAADRIA 2015).

[70] Lei H.Y., Li J.R., Xu Z.J., Wang Q.H. (2020). Parametric design of Voronoi-based lattice porous structures. Mater. Des..

[71] Peng W., Gonzalez-Ayala J., Guo J., Chen J., Hernández A.C. (2020). An alkali metal thermoelectric converter hybridized with a Brayton heat engine: Parametric design strategies and energetic optimization. J. Clean. Prod..

[72] Osyczka A., Gero J.S.B.T.-D.O. (1985). Multicriteria optimization for engineering design. Design Optimization.

[73] Modrak V., Soltysova Z. (2020). Batch size optimization of multi-stage flow lines in terms of mass customization. Int. J. Simul. Model..

[74] Milazzo M., Spezzaneve A., Persichetti A., Tomasi M., Peselli V., Messina A., Gambineri F., Aringhieri G., Roccella S. (2020). Digital and experimental synergies to design high-heeled shoes. Int. J. Adv. Manuf. Technol..

[75] Singh S., Singh G., Prakash C., Ramakrishna S. (2020). Current status and future directions of fused filament fabrication. J. Manuf. Process..

[76] Zhou L.-Y., Fu J., He Y. (2020). A review of 3D printing technologies for soft polymer materials. Adv. Funct. Mater..

[77] Barrios-Muriel J., Romero-Sánchez F., Alonso-Sánchez F.J., Rodríguez Salgado D. (2020). Advances in orthotic and prosthetic manufacturing: A technology review. Materials.

[78] Kromoser B., Pachner T. (2020). Optiknot 3D—Free-formed frameworks out of wood with mass customized knots produced by FFF additive manufactured polymers: Experimental investigations, design approach and construction of a prototype. Polymers.

[79] Modrak V., Soltysova Z. (2020). Management of product configuration conflicts to increase the sustainability of mass customization. Sustainability.

[80] Costa E.C.E., Jorge J., Knochel A.D., Duarte J.P. (2020). Enabling parametric design space exploration by non-designers. Artif. Intell. Eng. Des. Anal. Manuf..

[81] Zhao S., Zhang Q., Peng Z., Fan Y. (2020). Integrating customer requirements into customized product configuration design based on Kano’s model. J. Intell. Manuf..

[82] Jost P.J., Süsser T. (2020). Company-customer interaction in mass customization. Int. J. Prod. Econ..

[83] Dou R., Huang R., Nan G., Liu J. (2020). Less diversity but higher satisfaction: An intelligent product configuration method for type-decreased mass customization. Comput. Ind. Eng..

[84] Tookanlou P.B., Wong H. (2020). Determining the optimal customization levels, lead times, and inventory positioning in vertical product differentiation. Int. J. Prod. Econ..

[85] Martínez-Olvera C. (2020). An entropy-based formulation for assessing the complexity level of a mass customization industry 4.0 environment. Math. Probl. Eng..

[86] Kolarevic B. (2015). From mass customisation to design “democratisation”. Archit. Des..

[87] Yang L., Harrysson O.L.A., Cormier D., West H., Zhang S., Gong H., Stucker B. (2016). Design for additively manufactured lightweight structure: A perspective. The Solid Freeform Fabrication 2016, Proceedings of the 27th Annual International Solid Freeform Fabrication Symposium-An Additive Manufacturing Conference.

[88] Roger F., Krawczak P. 3D-printing of thermoplastic structures by FDM using heterogeneous infill and multi-materials: An integrated design-advanced manufacturing approach for factories of the future abstract. Proceedings of the 22ème Congrès Français de Mécanique.

[89] Feng J., Fu J., Lin Z., Shang C., Li B. (2018). A review of the design methods of complex topology structures for 3D printing. Vis. Comput. Ind. Biomed. Art.

[90] Orme M., Madera I., Gschweitl M., Ferrari M. (2018). Topology optimization for additive manufacturing as an enabler for light weight flight hardware. Designs.

[91] Suresh K. Efficient microstructural design for additive manufacturing. Proceedings of the ASME 2014 International Design Engineering Technical Conferences & Computers and Information in Engineering Conference.

[92] Saadlaoui Y., Milan J.-L., Rossi J.-M., Chabrand P. (2017). Topology optimization and additive manufacturing: Comparison of conception methods using industrial codes. J. Manuf. Syst..

[93] García-Domínguez A., Claver J., Sebastián M.A. (2017). Study for the selection of design software for 3D printing topological optimization. Procedia Manuf..

[94] Robert McNeel & Associates Rhinoceros. https://www.rhino3d.com/.

[95] Food4Rhino. https://www.food4rhino.com/.

[96] Barrios Hernandez C.R. (2006). Thinking parametric design: Introducing parametric gaudi. Des. Stud..

[97] Park H., Lee K.H. (2005). A new parametric control method for freeform mesh models. Int. J. Adv. Manuf. Technol..

[98] Sheffer A., Ungor A. (2001). Efficient adaptive meshing of parametric models. J. Comput. Inf. Sci. Eng..

[99] Fraile M. (2014). El nuevo paradigma contemporáneo. Del diseño paramétrico a la morfogénesis digital. Teor. Arquit. Contemponaneidad.

[100] Chang K.-H. (2015). Design Theory and Methods Using CAD/CAE.

[101] Wang W., Wang T.Y., Yang Z., Liu L., Tong X., Tong W., Deng J., Chen F., Liu X. (2013). Cost-effective printing of 3D objects with skin-frame structures. ACM Trans. Graph..

[102] Mahmoud D., Elbestawi M. (2017). Lattice structures and functionally graded materials applications in additive manufacturing of orthopedic implants: A review. J. Manuf. Mater. Process..

[103] Gibson L.J., Ashby M.F. (1997). Cellular Solids. Structure and Properties.

[104] García-Domínguez A., Claver J., Sebastián M.A. (2019). Infill optimization for pieces obtained by 3D printing. Procedia Manuf..

